# Modelling Photoionisation in Isocytosine: Potential Formation of Longer‐Lived Excited State Cations in its Keto Form

**DOI:** 10.1002/cphc.202100402

**Published:** 2021-09-07

**Authors:** Javier Segarra‐Martí, Michael J. Bearpark

**Affiliations:** ^1^ Department of Chemistry Molecular Sciences Research Hub Imperial College London White City Campus, 82 Wood Lane London W12 0BZ UK; ^2^ Present address: Instituto de Ciencia Molecular Universitat de Valencia P.O. Box 22085 ES-46071 Valencia Spain

**Keywords:** CASSCF/CASPT2, Conical Intersections, DNA/RNA, Photoionisation, Photostability

## Abstract

Studying the effects of UV and VUV radiation on non‐canonical DNA/RNA nucleobases allows us to compare how they release excess energy following absorption with respect to their canonical counterparts. This has attracted much research attention in recent years because of its likely influence on the origin of our genetic lexicon in prebiotic times. Here we present a CASSCF and XMS‐CASPT2 theoretical study of the photoionisation of non‐canonical pyrimidine nucleobase isocytosine in both its keto and enol tautomeric forms. We analyse their lowest energy cationic excited states including ${}^{2}\pi^{+}$ , ${}^{2}n_{O}^{+}$ and ${}^{2}n_{N}^{+}$ and compare these to the corresponding electronic states in cytosine. Investigating lower‐energy decay pathways we find – unexpectedly ‐ that keto‐isocytosine^+^ presents a sizeable energy barrier potentially inhibiting decay to its cationic ground state, whereas enol‐isocytosine^+^ features a barrierless and consequently ultrafast pathway analogous to the one previously found for the canonical (keto) form of cytosine^+^. Dynamic electron correlation reduces the energy barrier in the keto form substantially (by ∼1 eV) but it is nevertheless still present. We additionally compute the UV/Vis absorption signals of the structures encountered along these decay channels to provide spectroscopic fingerprints to assist future experiments in monitoring these intricate photo‐processes.

## Graphical abstract

**The photostability of isocytosine upon ionising radiation exposure** is assessed theoretically in both keto and enol tautomeric forms. This non‐canonical base, which features analogous UV excited state reactivity to DNA nucleobase cytosine, unexpectedly displays sizeable energy barriers along the cationic excited state in its keto form that hamper the decay. Simulations suggest isocytosine is less photostable against photoionisation, a factor that might contribute to selecting the more resilient cytosine under prebiotic VUV radiation exposure.

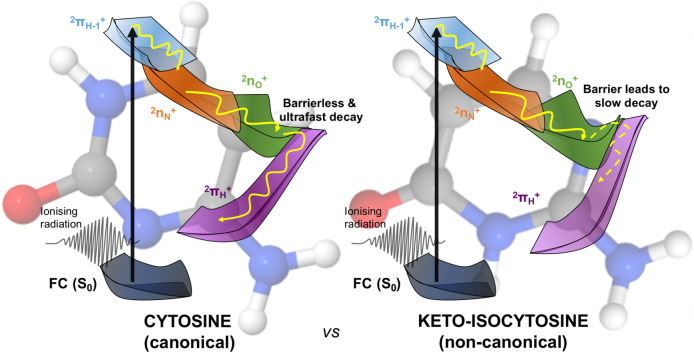

## 1 Introduction

DNA and RNA are known to significantly absorb UV/VUV light through their chromophoric species the nucleobases.[^1^, ^2^, ^3^] The excess energy gained can be quickly dissipated by means of ultrafast non‐radiative decays, which grant their photostability.^[4]^ However, such localised extra energy can also promote deleterious photochemical reactions, corrupting genetic material, and leading to healthcare concerns such as skin cancer melanoma.^[5]^

Vacuum UV (VUV) radiation is further known to trigger photoionisation in DNA aggregates, with electron removal generating radical cationic species[^6^, ^7^] which are reactive in the cellular environment, leading to damage causing apoptosis or cellular death.

The in‐depth study of nucleobase radical cations has so far been hampered (compared to photo‐excitation studies) because of the higher energies required for their formation:[^8^, ^9^, ^10^] first ionisation potentials at energies around 8 eV^[11]^ require intense VUV light sources for their generation *in vacuo*. However, additional recent interest follows evidence of significant ionisation yields in DNA/RNA nucleobases within complex double‐helix and guanine quadruplex structure motifs irradiated even with lower energy UV‐B light.[^12^, ^13^] This suggests that photoionisation can occur in certain circumstances when radiation under the monomer ionisation threshold is used, and that nucleobase radical cations might be formed in the cellular environment in larger yields than previously thought. Cytosine derivatives can also form their own type of aggregates (i‐motifs), which significantly alter photophysics,[^14^, ^15^] in this case mediated by charge separated or transfer states, and that are likely to involve the formation of cationic species.

**Figure 1:**
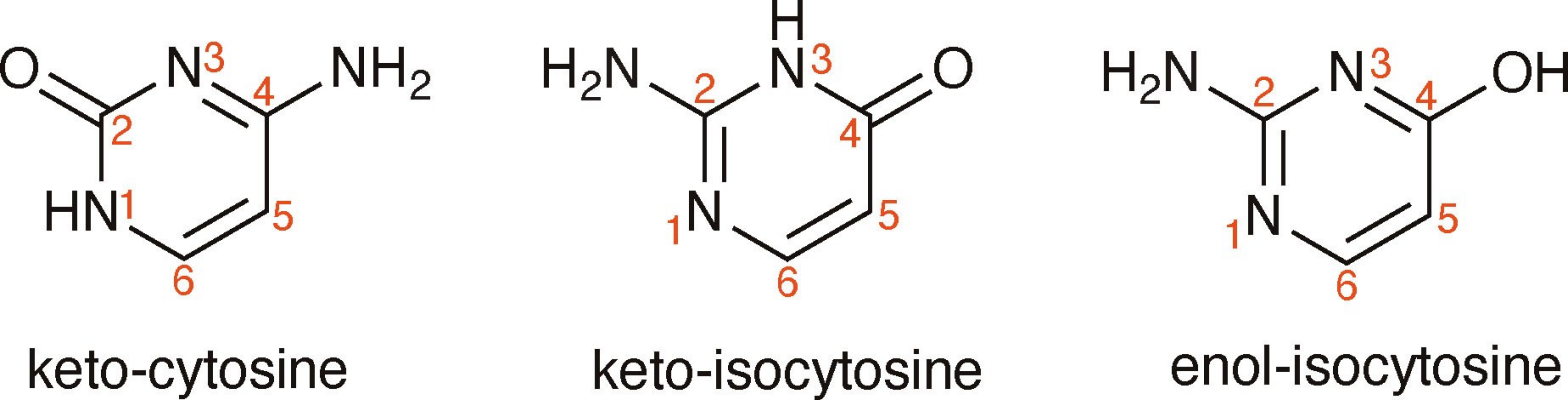
The molecular structure of keto and enol tautomers of isocytosine, together with their heteroatom labelling (in red).

An aspect hardly considered thus far is the photoionisation and subsequent relaxation of non‐canonical nucleobases, which are molecular species present in non‐negligible amounts in DNA.[^16^, ^17^] Excited state studies on non‐canonical nucleobases such as isocytosine,[^18^, ^19^, ^20^] an isomer of cytosine (see Figure 1), have attracted much less attention compared to their canonical counterparts and their behaviour remains largely unexplored to our knowledge despite its biological relevance.

Current thinking on DNA photophysics is that photostability was a necessary prerequisite for selecting the bases in prebiotic times under intense UV/VUV light exposure.[^21^, ^22^] This implies natural selection of the more resilient (photostable) building blocks to encode our genetic material and ensure the information was preserved and appropriately passed on. The vast majority of work has validated this scenario for UV light irradiation by studying both canonical and non‐canonical bases and their decays, but the role of the also present VUV radiation has so far been neglected. Building upon prior studies in the UV,[^19^, ^23^] we here explore the ability of the keto and enol forms of isocytosine to withstand VUV radiation (i. e. cation formation), in order to help understand their photostability in prebiotic times. We focus on isocytosine as it is a biologically relevant isomer of cytosine which can form Watson‐Crick base pairs with cytosine and isoguanine or reversed Watson‐Crick pairs with guanine in DNA.[^24^, ^25^]

Photostability is understood in this context as the ability of nucleobases to release the energy gained upon light absorption harmlessly following internal conversion on an ultrafast timescale. This rapid delocalisation of excess energy minimises the time spent by a chromophore in more reactive electronic excited states, thus reducing the formation of damaging photo‐products, even if the relationship between photo‐product formation and excited state lifetime is not always so clear cut.

It is worth noting that photostability is not the only criteria for selecting our genetic material. The selection of the underlying building blocks of DNA is not directly related to DNA's function, even if it is essential for providing the building blocks from which that function is derived. For instance, it is known that much of the current DNA function comes from the way it assembles into the double helix structure, which efficiently encodes and protects hereditary information. In this context, the exclusion of isocytosine from DNA/RNA central function has been ascribed to molecular evolution in which guanine was selected as it features only one stable tautomer in solution, thus being less prone to mispairings.^[26]^

Separately, interest in the dynamics of molecular cationic species has recently risen due to the advent of intense high‐energy light sources such as X‐ray free electron lasers (XFELs), which are capable of triggering photoionisation processes in organic molecular systems *in vacuo*.^[27]^ The possibility of studying photoionisations of DNA in the gas phase would allow us to gain insight into the different processes triggered upon higher‐energy light absorption, and motivates the present computational study.

In this article we study theoretically the cation electronic excited states in non‐canonical nucleobase isocytosine for both its keto and enol tautomeric forms. This is the first system for which we have made this comparison directly, both forms having been reported in the literature to be stable in the gas phase.^[28]^ Isocytosine is one of the many different non‐canonical instances that may feature in DNA and continues our work to assess theoretically the photoionisation of our genomic material.[^29^, ^30^] We start by considering the lowest‐lying ionisation potentials of keto‐ and enol‐isocytosine, which feature a range of accessible ${}^{2}\pi^{+}$ , ${}^{2}n_{O}^{+}$ and ${}^{2}n_{O}^{+}$ states at energies similar to those previously reported for cytosine.^[30]^ We then explore the fate of the different cationic electronic excited states of isocytosine^+^ once formed, producing an overall picture of the photo‐process that presents marked differences with respect to the canonical cytosine^+^: keto‐isocytosine^+^ presents a sizeable potential energy barrier between the first excited cationic ${}^{2}n_{O}^{+}$ state and the ground state which is heavily reliant on dynamic electron correlation for its accurate description, whereas enol‐isocytosine^+^ – despite featuring marked structural differences – presents a range of accessible conical intersections similar to cytosine, leading to an ultrafast decay to the ground state. We additionally predict UV/Vis spectra at relevant key structures, paving the way to monitor these photo‐processes experimentally in the near‐future.

## Computational Details

The OpenMOLCAS[^31^, ^32^, ^33^] electronic structure theory package was used for most of the computations reported. An atomic natural orbital basis set (ANO−L) was used throughout in its valence double‐*ζ* polarised contraction.[^34^, ^35^] The active space for (keto)‐isocytosine comprises the full *π* valence occupied and virtual space plus the two occupied *n* lone pair (*n_O_* and *n_N_*) orbitals to include the ${}^{2}n_{O}^{+}$ and ${}^{2}n_{O}^{+}$ states, totalling 14 electrons in 10 orbitals for the neutral and 13 electrons in 10 orbitals for the cationic species. For enol‐isocytosine, a similar active space was employed: we included all *π* valence occupied orbitals except the one localised on the amino moiety, due to its elevated (1.99) occupation number, all virtual *π* orbitals, and the two occupied *n_N_*) pairs to include ${}^{2}n_{O}^{+}$ states, resulting in 12 electrons in 9 orbitals for the neutral and 11 electrons in 9 orbitals for the cationic species.

CASSCF wave functions were averaged over five doublet states and were subsequently used for single‐point CASPT2 energy corrections.[^36^, ^37^, ^38^] An imaginary level shift of 0.2 a.u. was employed in the perturbative step to avoid the presence of intruder states,^[39]^ and IPEA shifts^[40]^ of 0.0 and 0.25 a.u. were tested as this correction has been shown to improve the description of cationic open‐shell states in these systems.^[11]^

CASPT2 computations were performed in its single‐state,[^36^, ^37^, ^38^] multistate (MS),^[41]^ and extended multistate (XMS)^[42]^ variants to benchmark the effect of the zeroth order Hamiltonian on the cationic manifold. For presenting and discussing the energies at the Franck‐Condon (FC) region, we have chosen to average over the different CASPT2 formulations as this allows us to show the mean value as well as the standard deviation expected by modifying the zeroth‐order Hamiltonian. However, for geometries and energies away from the FC region we have only reported XMS‐CASPT2 estimates as this has been shown to provide a better balance in the simultaneous description of covalent and ionic excited states^[42]^ and therefore gives us more reliable estimates, particularly at or nearby crossing regions.[^43^, ^44^]

The resolution of identity based on the Cholesky decomposition was used to speed up the calculation of the electron repulsion integrals,[^45^, ^46^, ^47^] and was used for both energy evaluations^[48]^ as well as in calculating CASSCF analytical gradients[^49^, ^50^] and non‐adiabatic couplings.^[51]^ Second‐order nuclear derivatives were computed numerically employing the aforementioned gradients.^[52]^ CASSCF conical intersections (CIs) were characterised with the method of Fdez Galván et al.^[51]^

The characterised cationic ground and excited state minima, as well as the different low‐lying CIs were also optimised at the CASPT2 level of theory to uncover the role of dynamic electron correlation on the optimised geometries, as it is known to heavily impact those in the singlet manifold.[^53^, ^54^, ^55^] CASPT2 minima and CI optimisations (using the projection method of Bearpark *et al*.^[56]^) were carried out with analytical gradients[^57^, ^58^, ^59^] and couplings^[60]^ as implemented in BAGEL.[^61^, ^62^]

Additional simulations averaging over the lowest‐lying 30 electronic doublet states were carried out on top of the different characterised minima to evaluate ground and excited state absorption signals.[^63^, ^64^] We have assumed that excited state absorption of the individual ${}^{2}n^{+}$ and ${}^{2}\pi^{+}$ states are dominated by the electronic structure at their corresponding minima,[^65^, ^66^] thus neglecting the time‐evolution of the system and its lineshape, which is costly to simulate and out of the scope of the present study.[^67^, ^68^, ^69^, ^70^, ^71^] The CAS state interaction method^[72]^ was used to evaluate transition dipole moments and oscillator strengths and energies were corrected with the standard (single‐state) CASPT2 formulation with an IPEA shift of 0.0. The transitions so obtained were broadened with Gaussian functions with full width at half maximum of 0.3 eV, as used in similar organic systems.^[73]^ Ground and excited state absorption signals were broadened as implemented in Gabedit^[74]^ and orbital visualisation was performed with Molden.^[75]^

## 2 Results

The results are divided into three sections: first the ionisation potentials of keto and enol derivatives of isocytosine are computed and compared to those previously obtained for cytosine,^[30]^ which allows us to select the level of theory used throughout the rest of the study; then the different excited state decay pathways of keto and enol isocytosine^+^ are investigated by characterising their respective excited state minima and interstate crossings; finally UV/Vis spectra are computed on top of well‐defined ground and excited state cationic minima in order to provide a route map for experiments.

### 2.1 Ionisation Potentials

We start by looking at the computed lowest‐lying ionisation potentials displayed by the keto and enol forms of isocytosine compared to cytosine.^[30]^

**Figure 2:**
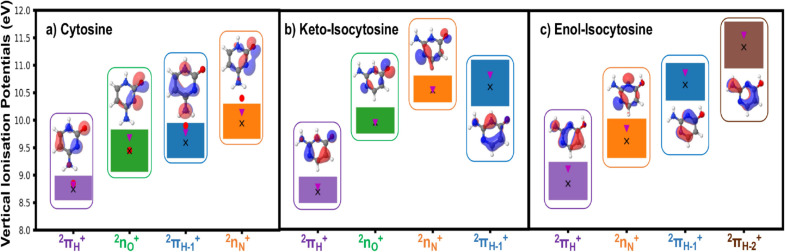
Schematic representation of the gas phase vertical ionisation potentials of a) cytosine^[30]^ b) keto‐isocytosine and c) enol‐isocytosine, computed with a range of zeroth‐order CASPT2 Hamiltonians. This scheme provides the range of ionisation potential estimates spanned by the different zeroth‐order Hamiltonians, where ${}^{2}\pi_{H}^{+}$ is depicted as squares in purple, ${}^{2}n_{O}^{+}$ in green, ${}^{2}\pi_{H-1}^{+}$ in blue, ${}^{2}n_{N}^{+}$ in orange and ${}^{2}\pi_{H-2}^{+}$ in brown. The specific values for each CASPT2 formulation are provided in Tables S3 and S4 in the SI. CASPT2 averages are represented by black crosses, red dots denote th*e* experimental evidence available for cytosine and given for comparison^[76]^ and magenta inverted triangles represent the estimates at the XMS‐CASPT2(IPEA=0.0) level of theory used in section 3.2. The singly occupied molecular orbitals (SOMOs) characterising the different cationic states are also given.

Figure 2 displays the different computed vertical ionisation potentials in keto (panel b) and enol (panel c) isocytosine, together with their comparison with earlier calculations on cytosine (panel a) for which there is also experimental data. The first ionisation potential of both keto and enol forms of isocytosine (and cytosine itself)^[30]^ is characterised by an unpaired electron in the singly occupied molecular orbital (SOMO) corresponding to the *π_H_* orbital (see Figure 2, purple), leading to the ${}^{2}\pi_{H}^{+}$ cationic state. The averaged ionisation potential for this lowest‐lying cationic state is 8.68 and 8.83 eV, respectively, for the keto and enol tautomers, with a standard deviation of 0.15 and 0.37 eV. These are comparable to the 8.74 eV value and 0.16 eV standard deviation previously obtained for cytosine.^[30]^

The next state for all forms corresponds to a lone pair ionisation, which is depicted by ${}^{2}n_{O}^{+}$ (Figure 2, green) and ${}^{2}n_{N}^{+}$ (Figure 2, orange) states for keto and enol forms, respectively, and which are placed at 9.95 and 9.61 eV adiabatically from the ground state with a standard deviation of 0.13 and 0.36 eV. Due to the presence of the OH group, the enol tautomer does not feature a second lone pair. On the other hand, keto‐isocytosine displays a ${}^{2}n_{N}^{+}$ excitation placed at 10.54 eV with a standard deviation of 0.16. These states are comparable to the estimates recorded for cytosine of 9.44 and 9.94 eV for the ${}^{2}n_{O}^{+}$ and ${}^{2}n_{N}^{+}$ states, respectively.

The last states to analyse are characterised by an unpaired electron in the ${}^{2}\pi_{H-1}^{+}$ SOMO, and which is placed adiabatically at 10.60, 10.64 and 9.59 eV, for keto‐, enol‐isocytosine and cytosine, respectively. These last states show a clear blue‐shift (∼1 eV) in isocytosine with respect to cytosine, featuring also much larger standard deviations of 0.49 and 0.34 eV for keto and enol forms as displayed in Figure 2. The fourth state in enol‐isocytosine, the only system not featuring a second lone pair ${}^{2}n_{O}^{+}$ state, is of ${}^{2}\pi_{H-2}^{+}$ character and is placed at 11.33 eV with a large standard deviation of almost half an eV (0.49), being placed at considerably higher energies than any of the other states considered.

It is worth noting that whereas clear similarities can be drawn between the SOMOs responsible for ${}^{2}\pi_{H}^{+}$ , ${}^{2}n_{O}^{+}$ and ${}^{2}n_{N}^{+}$ across the different cytosine isomers considered, ${}^{2}\pi_{H-1}^{+}$ appears to be less consistent: the SOMO depicting this orbital is clearly analogous in the keto forms of isocytosine and cytosine but it appears to deviate slightly more when considering enol‐isocytosine. In any case, the differences between the SOMOs are not critical and the vertical ionisation potentials computed seem to be analogous for both isocytosine systems, which makes us consider them under the same state labelling.

The IPEA shift correction in CASPT2 has been tested with values of 0.0 and of 0.25 a.u. The former means no correction is included, whereas the latter is the default value obtained in the original implementation of the method and that yields the best estimates to reproduce diatomic dissociation energies,^[40]^ which is how the technique was calibrated. More recent studies have looked at how this shift influences vertical excitation energies, displaying a slight dependence on system and basis set size.^[77]^ Our interest in this magnitude hinges from previous studies,^[11]^ which showed that the inclusion of IPEA shift improved the agreement with the recorded vertical ionisation energies.

Tables S3 and S4 show how the inclusion of an IPEA shift systematically blue‐shifts the ionisation energies. This helps single‐state (SS)‐CASPT2 estimates approach the experimental reference values as has been shown elsewhere,^[11]^ but it leads to important over‐estimations when combined with either multistate (MS) or extended multistate (XMS) variants. Because we are using XMS‐CASPT2 to map potential energy surfaces, we therefore use an IPEA shift of 0.0 a.u. as this combination provides the most accurate energies for studying decay pathways.

We analyse the adiabatic ionisation potentials next, which are reported in Table 1. These magnitudes show lesser dispersion in the estimates obtained for the different methods employed here, displaying a ∼0.1 eV red‐shift and blue‐shift for keto and enol isocytosine, respectively, in comparison to cytosine. We have also explored the potential role of dynamic electron correlation in the nuclear geometries optimised and how this may shift the estimates, obtaining a small ∼0.1 eV red‐shift in all cases, which is consistent with previous studies measuring this difference for the photo‐excitation of adenine.^[78]^ Re‐optimisation has not significantly changed these values; CASPT2 is necessary for describing correct energy differences whereas it appears to be less important for describing these geometries themselves.[^29^, ^30^]

**Table 1:** Adiabatic ionisation potentials (in eV) of the lowest‐lying ${}^{2}\pi_{H}^{+}$ state for cytosine, keto and enol isocytosine computed with different zeroth‐order CASPT2 Hamiltonians, the IPEA values given in a.u. The reference (${}^{2}\pi_{H}^{+}$ )_min_ geometry is taken at the CASSCF level of theory (estimates for XMS‐CASPT2 geometries in parenthesis).

		Cytosine	Keto‐Isocytosine	Enol‐Isocytosine
CASPT2	IPEA=0.0	8.34 (8.21)	8.24 (8.12)	8.39 (8.25)
	IPEA=0.25	8.50 (8.38)	8.40 (8.30)	8.52 (8.40)
MS‐CASPT2	IPEA=0.0	8.44 (8.30)	8.30 (8.17)	8.52 (8.38)
	IPEA=0.25	8.57 (8.45)	8.46 (8.35)	8.64 (8.51)
XMS‐CASPT2	IPEA=0.0	8.72 (8.54)	8.65 (8.51)	8.94 (8.80)
	IPEA=0.25	8.88 (8.72)	8.80 (8.68)	9.05 (8.93)
CASPT2 Average		8.56 (8.43)	8.48 (8.36)	8.68 (8.55)

Overall, we can conclude that the different isomers of cytosine studied here display a very similar first ionisation potential, characterised by a ${}^{2}\pi_{H}^{+}$ cationic state. The two keto forms studied (of cytosine and isocytosine), present also comparable ${}^{2}n_{O}^{+}$ and ${}^{2}n_{N}^{+}$ states, while differing substantially in the ${}^{2}\pi_{H-1}^{+}$ state, which is also swapped in energetic order, i. e. it features as *D*_2_ in cytosine and as *D*_3_ in isocytosine. Enol‐isocytosine, on the other hand, presents an analogous ${}^{2}n_{N}^{+}$ state, slightly red‐shifted with respect to the keto tautomer, and a ${}^{2}\pi_{H-1}^{+}$ state comparable to that of its keto form, even if the SOMO characterising this transition may display more marked differences.

Unfortunately there are no experimental measurements available to our knowledge for the vertical ionisation energies of isocytosine, in either keto or enol forms, and it is therefore difficult to assess which CASPT2 Hamiltonian would be more appropriate to model these cationic species when considering their decay. We decided to use XMS‐CASPT2(IPEA=0.0), firstly because it shows the best agreement in cytosine (an isomeric form of isocytosine)^[30]^ and it has also been reported to be more appropriate for describing potential energy crossing regions,^[43]^ which are relevant for photochemical studies.

Moreover, based on the simulations discussed above, XMS‐CASPT2(IPEA=0.0) shows the closest agreement (magenta inverted triangles in Figure 2) with the available experimental data, and hence has been used for the study of the potential energy surfaces described in the following section.

### 2.2 Excited State Decay Pathways

Upon strong field (ionising) radiation exposure, DNA nucleobases have been shown to form a variety of cationic species,[^10^, ^79^] as opposed to what is often observed in the singlet manifold where a given dipole‐allowed transition gathers most of the oscillator strength.^[1]^ This means one needs to consider several different starting states (or linear combinations of them) and how they all decay in order to understand this complex photo‐process. To do this, we assume direct population to the highest state considered in this work (${}^{2}\pi_{H-1}^{+}$ for keto and ${}^{2}\pi_{H-2}^{+}$ for enol), as that allows us to explore all lower‐lying states and their particular roles in the excited state deactivation down to the cationic ground (${}^{2}\pi_{H}^{+}$ ) state.

#### 2.2.1 Keto‐isocytosine^+^

**Figure 3:**
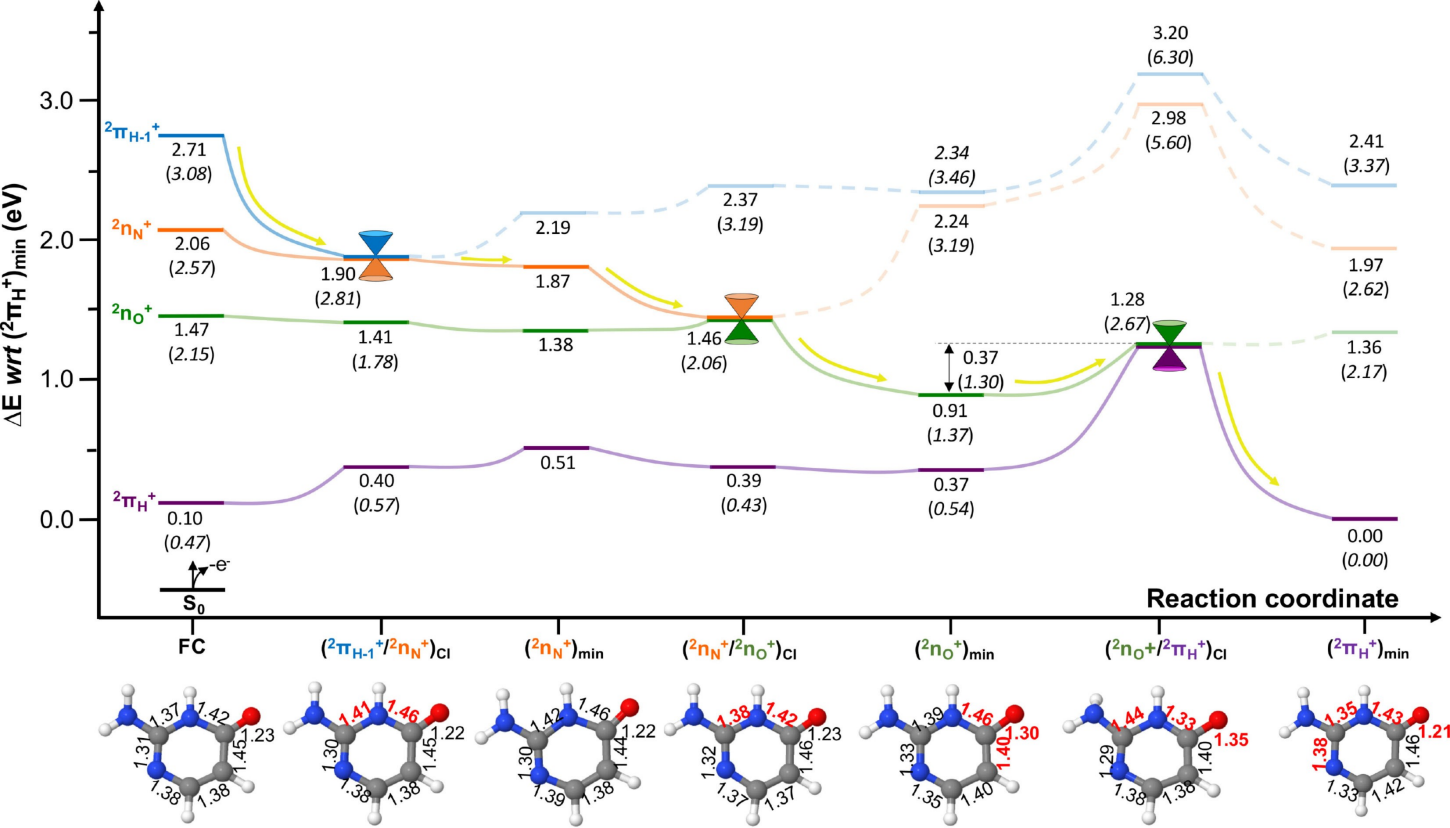
Potential energy surfaces of cationic keto isocytosine computed at the XMS‐CASPT2(IPEA=0.0) level of theory (CASSCF energy values at CASSCF geometries in parenthesis). All energies are given in eV with respect to (${}^{2}\pi_{H}$ )_*min*_. Yellow arrows represent the evolution of the excited state population assuming initial activation of the ${}^{2}\pi_{H-1}^{+}$ state. The XMS‐CASPT2 characterised critical structures are provided along the potential energy surface to highlight the main nuclear displacements embodying this photo‐reaction, with the main bond length distances (in Å) given in red. A comparison between the XMS‐CASPT2 and CASSCF optimised structures is provided in the SI (Figure S1).

Figure 3 displays the cationic excited state decay of keto‐isocytosine^+^ following initial ionisation to the ${}^{2}\pi_{H-1}^{+}$ state, which is depicted by yellow arrows throughout the potential energy surface diagram. This leads to a barrierless relaxation to the ${{(}^{2}\pi_{H-1}^{+}{/}^{2}n_{N}^{+})}_{CI}$ , which entails a ∼0.04 Å stretch of both C2‐N3 and N3‐C4 bonds (see Figure 1 for atom labelling), and that funnels the population down to the ${}^{2}n_{N}^{+}$ state.

Once populating the ${}^{2}n_{N}^{+}$ state, further relaxation leads to the (${}^{2}n_{N}^{+}$ )_*min*_ structure, which we were unable to characterise at the CASSCF level of theory. This shallow minimum lies very close in energy to ${{(}^{2}\pi_{H-1}^{+}{/}^{2}n_{N}^{+})}_{CI}$ and features a very similar structural motif. Further relaxation leads barrierlessly to the ${{(}^{2}n_{N}^{+}{/}^{2}n_{O}^{+})}_{CI}$ , which is characterised by a bond length shortening of 0.04 Å of C2‐N3 and N3‐C4 bonds, recovering a similar bond length pattern to that observed at the Franck‐Condon (FC) equilibrium region.

Upon reaching ${{(}^{2}n_{N}^{+}{/}^{2}n_{O}^{+})}_{CI}$ the excited state population is transferred to the ${}^{2}n_{O}^{+}$ state, which further relaxes to (${}^{2}n_{O}^{+}$ )_*min*_ with pronounced structural rearrangements around the carbonyl C4‐O moiety. Concretely, we observe a C4‐O and N3‐C4 bond lengthening of 0.07 and 0.04 Å, and a C4‐C5 bond shortening of 0.06 Å.

Once populating the ${}^{2}n_{O}^{+}$ state, further relaxation is hampered by a potential energy surface barrier, which requires an additional 0.37 eV to be surmounted to reach the ${{(}^{2}n_{O}^{+}{/}^{2}\pi_{H}^{+})}_{CI}$ that mediates the non‐radiative decay to the ground state. The main structural parameters responsible at the XMS‐CASPT2 level for the ${{(}^{2}n_{O}^{+}{/}^{2}\pi_{H}^{+})}_{CI}$ are an elongation of the already stretched carbonyl C4‐O bond and the C2‐N3 bond of 0.05 Å, and a very pronounced N3‐C4 bond length shortening of 0.13 Å. This was the lowest energy point we could find in the intersection seam, and is markedly different to what we have previously found for other canonical pyrimidine nucleobases upon ionisation, which display barrierless and ultrafast decays to the cationic ground state.[^29^, ^30^]

Furthermore, upon inspection of the relative CASSCF energies provided in Figure 3, it can be seen how the potential energy barrier with this less correlated method requires more energy (1.30 eV) to be surmounted. This larger potential energy barrier observed at CASSCF with respect to XMS‐CASPT2 is associated to the different geometries obtained with both methods: whereas XMS‐CASPT2 induces a twist in the amino moiety, as well as a ∼5° out‐of‐plane puckering in the main heterocyclic frame, CASSCF appears to not recover enough correlation to induce the aforementioned out‐of‐plane motions[^53^, ^54^, ^55^] and is forced to further elongate and bend the carbonyl group instead (see SI Figure S1) increasing the energy barrier. This highlights the importance of treating dynamic electron correlation effects on both energies and geometries when establishing decay mechanisms.

With sufficient energy to access ${{(}^{2}n_{O}^{+}{/}^{2}\pi_{H}^{+})}_{CI}$ , a swift ${}^{2}n_{O}^{+}$ →${}^{2}\pi_{H}^{+}$ population transfer is expected with subsequent relaxation to the (${}^{2}\pi_{H}^{+}$ )_*min*_ cationic ground state. This is encompassed by a substantial bond length redistribution across most of the molecular frame, but mostly on the N1‐C2, C2‐N3, N3‐C4 and C4‐O bonds (see Figure 3).

Overall, we predict an initial very rapid ${}^{2}\pi_{H-1}^{+}\to^{2}n_{N}^{+}\to^{2}n_{O}^{+}$ excited state relaxation in the keto tautomer of isocytosine^+^, with a longer‐lived component associated to the final ${}^{2}n_{O}^{+}\to^{2}\pi_{H}^{+}$ part of the decay that is hampered by a sizeable potential energy barrier connecting the ${}^{2}n_{O}^{+}$ minimum and the ${{(}^{2}n_{O}^{+}{/}^{2}\pi_{H}^{+})}_{CI}$ facilitating population transfer to the ${}^{2}\pi_{H}^{+}$ cationic ground state. Importantly, the lifetime expected of this latter component varies depending on the electron correlation retained in the model, and this points to the necessity of using strongly correlated (both static and dynamic) approaches to properly model this photo‐reaction. This differs from canonical nucleobase and isomer keto‐cytosine,^[30]^ which presents a barrierless and ultrafast decay to the ground state that facilitates the rapid funneling of the excess energy gained upon photo‐ionisation to the cationic ground state, and where the effects of dynamic electron correlation were found to be much less pronounced.

#### 2.2.2 Enol‐isocytosine^+^

**Figure 4:**
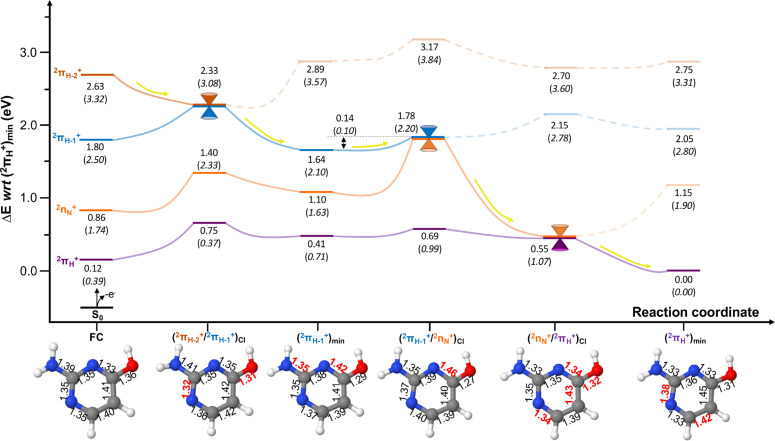
Potential energy surfaces of cationic enol isocytosine computed at the XMS‐CASPT2(IPEA=0.0) level of theory (CASSCF energy values at CASSCF geometries in parenthesis). All energies are given in eV with respect to (${}^{2}\pi_{H}^{+}$ )_*min*_. Yellow arrows represent the evolution of the excited state population assuming initial activation of the ${}^{2}\pi_{H-2}^{+}$ state. The XMS‐CASPT2 optimised critical structures are provided along the potential energy surface to highlight the main nuclear displacements in subsequent structures, with the main deviations in bond length distances (in Å) highlighted in red. A comparison between the XMS‐CASPT2 and CASSCF optimised structures is provided in the SI (Figure S2).

Photoionisation relaxation pathways of the enol form of isocytosine^+^ are reported in Figure 4. As previously mentioned, enol‐isocytosine does not have a *n_O_* lone pair and thus does not feature a ${}^{2}\pi_{H}^{+}$ state, and for this reason we have decided to include an additional higher‐lying ${}^{2}\pi_{H-2}^{+}$ cationic state.

Upon population, the dissociative ${}^{2}\pi_{H-2}^{+}$ state relaxes directly to the ${{(}^{2}\pi_{H-2}^{+}{/}^{2}\pi_{H-1}^{+})}_{CI}$ , which displays pronounced N1‐C2 and C4‐O bond length shortenings of 0.03 and 0.05 Å, respectively, and that facilitates population transfer to the ${}^{2}\pi_{H-1}^{+}$ state. It is worth noting that this structure is strongly affected by the inclusion of dynamic electron correlation in the model, showcasing a C6‐N1‐C2‐N3 out‐of‐plane dihedral of 12.3° resulting in a puckered N3 atom. Further details on this structure and a more thorough comparison with the planar structure obtained with CASSCF are provided in the SI.

The ${}^{2}\pi_{H-1}^{+}$ state relaxes to its minimum (${}^{2}\pi_{H-1}^{+}$ )_*min*_ that entails a marked ∼0.7 eV stabilisation encompassed by a shortening of the C2‐N and a lengthening of the N3‐C4 bonds by 0.06 and 0.07 Å respectively that aid recovering the planarity of the heterocyclic molecular frame. From this minimum, a 0.14 eV barrier has to be surmounted in order to reach the ${{(}^{2}\pi_{H-1}^{+}{/}^{2}n_{N}^{+})}_{CI}$ that mediates the population of the ${}^{2}n_{N}^{+}$ state, which has an associated increase in the N3‐C4 bond length by 0.04 Å. In contrast with the keto isomer, enol‐isocytosine displays analogous energy barriers at both CASSCF and XMS‐CASPT2 levels of theory and is thus expected to be less reliant on the dynamic electron correlation included in the model.

Once reaching ${{(}^{2}\pi_{H-1}^{+}{/}^{2}n_{N}^{+})}_{CI}$ the population is funnelled to the ${}^{2}n_{N}^{+}$ state, which does not feature a minimum and descends a further 1.23 eV to reach the ${{(}^{2}n_{N}^{+}{/}^{2}\pi_{H}^{+})}_{CI}$ that mediates the decay to the cationic ${}^{2}\pi_{H}^{+}$ ground state. This conical intersection is characterised by pronounced structural changes around the C5‐hydroxyl group and encompasses very marked 0.12 Å N3‐C4 and a 0.06 Å C6‐N1 bond length shortenings and C4‐O and C4‐C5 bond lengthenings of 0.05 and 0.03 Å, respectively.

After accessing ${{(}^{2}n_{N}^{+}{/}^{2}\pi_{H}^{+})}_{CI}$ the population is then transferred to the ${}^{2}\pi_{H}^{+}$ ground state, which undergoes a further ∼0.5 eV energy relaxation to its (${}^{2}\pi_{H}^{+}$ )_*min*_ structure that encompasses the elongation of the C5‐C6 and N1‐C2 bonds by 0.03 Å.

In summary, we expect an ultrafast ${}^{2}\pi_{H-2}^{+}\to^{2}\;\pi_{H-1}^{+}\to^{2}\;n_{N}^{+}\to^{2}\;\pi_{H}^{+}$ excited state relaxation of the enol tautomer of isocytosine^+^, given the negligible barriers presented along the decay. Dynamic electron correlation appears to influence mostly the ${{(}^{2}\pi_{H-2}^{+}{/}^{2}\pi_{H-1}^{+})}_{CI}$ placed in the vicinity of the Franck‐Condon region, leading to an out‐of‐plane puckered structure, which however does not influence the qualitative picture of the decay with respect to that obtained with CASSCF.

The ultrafast character predicted for enol‐isocytosine^+^ resembles more that of cytosine^+^ than its keto tautomeric form described above. It is important to note that potential energy barriers are found in both cases along the decay: a small ∼0.1 eV connecting the ${(}^{2}\pi_{H-1}^{+}$ )_*min*_→${{(}^{2}\pi_{H-1}^{+}{/}^{2}n_{N}^{+})}_{CI}$ intermediate step in enol‐isocytosine^+^, which is expected to still lead to ultrafast decays, and a more sizeable 0.3 eV connecting the (${}^{2}n_{O}^{+}$ )_*min*_→${{(}^{2}n_{O}^{+}{/}^{2}\pi_{H}^{+})}_{CI}$ final decay step in keto‐isocytosine^+^, which we predict to be the rate‐limiting step. Another important aspect to consider is that the initial energy gap spanned by the low‐lying cationic state manifold of isocytosine is significantly larger than that of cytosine (∼2.6/2.5 for keto/enol *vs* ∼2 eV).^[30]^ We expect these two highlighted aspects to have implications for future molecular dynamics simulations, both in terms of nuclear (lifetimes for relaxation to the cationic ground state) and electron (initial coherences formed upon absorption) dynamics, which we are currently investigating.

It is important to note that the reaction paths depicted in this section refer to the initial formation of a *D*_3_ excited cationic state (${}^{2}\pi_{H-1}^{+}{/}^{2}\pi_{H-2}^{+}$ for keto/enol tautomers, respectively), and which conditions the reaction coordinates triggered thereafter. The potential energy barriers shown by the *D*_0_ state in Figures 3 and 4 between the FC region and ${{(}^{2}\pi_{H}^{+})}_{min}$ are the energy changes of this state associated with geometry changes determined by excited states. These pathways will not be followed on the *D*_0_ state alone: direct access to *D*_0_ (${}^{2}\pi_{H}^{+}$ ) leads to barrierless relaxation to ${{(}^{2}\pi_{H}^{+})}_{min}$ .

### 2.3 UV/Vis Spectroscopy

In order to aid experiments in the characterisation of these complex photo‐processes, we provide next the estimates for UV/Vis spectral signals expected to arise from the different optimised ground and excited state cationic minima. The results rely on the assumption that both ground or excited state absorption of each individual minima are dominated by their specific electronic state,^[66]^ and are broadened phenomenologically with Gaussian functions (see Computational details).[^54^, ^80^]

**Figure 5:**
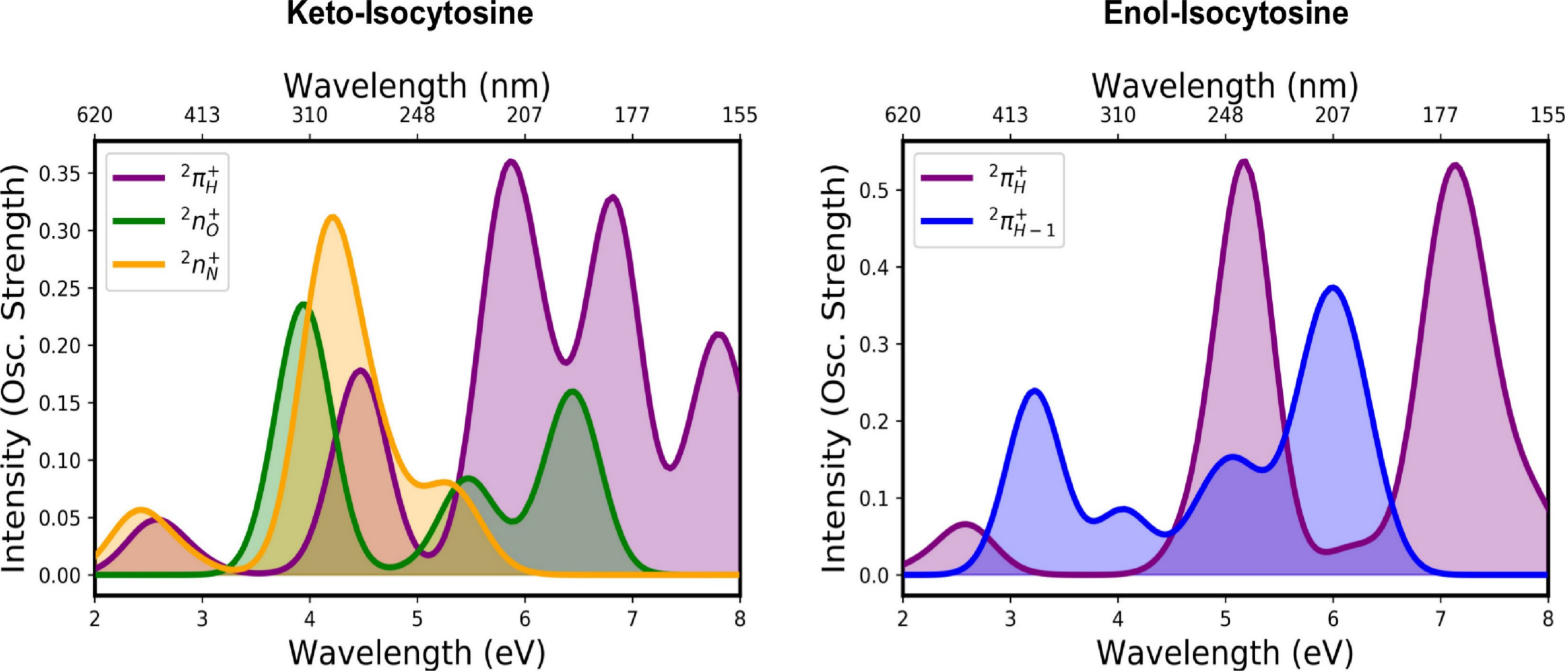
Electronic absorption signals of cationic keto‐isocytosine (left‐hand side) and enol‐isocytosine (right‐hand side) in their ground (${}^{2}\pi_{H}^{+}$ ) and excited state (${}^{2}n_{O}^{+}$ /${}^{2}n_{N}^{+}$ /${}^{2}\pi_{H-1}^{+}$ ) minima, as shown in Figures 3 and 4 and calculated with the XMS‐CASPT2(IPEA=0.0) level of theory.

Figure 5 displays the ground and excited state UV/Vis absorption features for keto and enol isocytosine^+^, respectively. As can be seen, both keto and enol forms of isocytosine present significant absorption features throughout the 200–600 nm range (2 to 6 eV), and corresponding mostly to those associated to the cationic ground state state ${}^{2}\pi_{H}^{+}$ . Keto (left‐hand panel) and enol (right‐hand panel) tautomers present different excited state minima, corresponding to ${}^{2}n_{O}^{+}$ and ${}^{2}n_{N}^{+}$ cationic states for the former and the ${}^{2}\pi_{H-1}^{+}$ state for the latter, respectively.

For keto‐isocytosine^+^, signals from ${}^{2}n^{+}$ states appear mostly in the 3–5 eV energy range and feature significant intensities (larger than those coming from ${}^{2}\pi_{H}^{+}$ ) around 300 nm and that is particularly noticeable for the ${}^{2}n_{N}^{+}$ ${}^{2}n_{N}^{+}$ state. Enol‐isocytosine^+^, on the other hand, present signals arising only from ${}^{2}\pi^{+}$ states, and which display sizeable intensities in different parts of the spectral region: a significant signal at ∼400 nm and a more intense peak at ∼200 nm in‐between the main absorbing features of the ${}^{2}\pi_{H}^{+}$ ground state.

Despite the congested spectral signals predicted for the 200–600 nm probing window, we expect certain regions to feature fingerprints arising from only one of the cationic electronic states that may enable a state‐specific monitoring of these photo‐reactions as it has also been postulated for the canonical pyrimidine nucleobases.[^29^, ^30^] In both keto and enol tautomers we observe an absorption signal in the 400–600 (2 to 3 eV) spectral region that is associated mostly to the ${}^{2}\pi_{H}^{+}$ cationic ground state. On the other hand, ${}^{2}n^{+}$ states present distinctive absorption features around 300, appearing in separated enough wavelengths to be able to target them separately, and the ${}^{2}\pi_{H-1}^{+}$ state of enol‐isocytosine that peaks at ∼400 nm in the low energy window.

Both $D_{0}{(}^{2}\pi_{H}^{+})$ and $D_{1}{(}^{2}n_{O}^{+}{/}^{2}\pi_{H-1}^{+})$ or $D_{2}{(}^{2}n_{N}^{+})$ spectral signals are included in the present study, the former referring to ground state absorptions and the latter two to transient signals that may be registered in the different electronic excited states. The different spectral regions in which these two states are predicted to absorb should help future experiments monitoring photoionisation processes, as it helps depict a state‐to‐signal assignment that aids in the interpretation of photo‐processes as has been shown elsewhere.^[81]^

Overall, we can conclude the UV/Vis window appears to be a suitable probe region for keto‐isocytosine in order to monitor these intricate photo‐processes, as it provides more separated signals as was previously observed for isomer cytosine.^[30]^ The enol form of isocytosine, on the other hand, shows a more congested UV/Vis window that nevertheless features a signal at ∼400 nm that may enable monitoring the ${}^{2}\pi_{H-1}^{+}$ state separately. Whereas the spectra associated to the ${}^{2}\pi_{H}^{+}$ state appears to be similar and shifted for keto and enol isocytosine, the ${}^{2}n^{+}$ states lead to larger differences mostly due to being of different character (*n_O_* for the keto and *n_N_* for the enol forms).

## 3 Conclusions

In this work the photoionisation phenomena triggered in non‐canonical nucleobase isocytosine in both its keto and enol tautomeric forms upon VUV light absorption is studied for the first time. We investigate the effect of high‐energy radiation on less studied but still important isomeric DNA systems of biological relevance, which showcases relaxation pathways leading cationic excited state populations to the ground state in predicted ultrafast timescales, similar to the well‐known deactivation of canonical DNA/RNA nucleobases in singlet states which is suggested to be central to their photostability.[^1^, ^4^, ^21^, ^22^]

The characterised XMS‐CASPT2 potential energy surfaces show different behaviors for keto and enol tautomeric forms of isocytosine^+^, as well as being significantly different to the previously characterised isomer cytosine.^[30]^

For keto‐isocytosine^+^ we predict an ultrafast ${}^{2}\pi_{H-1}^{+}\to^{2}n_{N}^{+}\to^{2}n_{O}^{+}$ decay with a kinetic limiting ${}^{2}n_{O}^{+}\to^{2}\pi_{H}^{+}$ step heavily controlled by electron dynamic correlation and out‐of‐plane motions, not observed in other cationic DNA related systems thus far.[^29^, ^30^] This is unexpected, as the isomer cytosine presents a barrierless profile,^[30]^ and considering that both cytosine and isocytosine were recently shown to display analogous ultrafast excited state singlet decays upon UV light photo‐excitation due to their strong resemblance.^[19]^

Upon accessing the highest‐lying ${}^{2}\pi_{H-1}^{+}$ state of the keto form, the excited state population will reach ${{(}^{2}\pi_{H-1}^{+}{/}^{2}n_{N}^{+})}_{CI}$ populating the ${}^{2}n_{N}^{+}$ state minimum. ${{(}^{2}n_{N}^{+})}_{min}$ features a close‐lying and accessible ${{(}^{2}n_{N}^{+}{/}^{2}n_{O}^{+})}_{CI}$ that mediates the population transfer and relaxation to ${{(}^{2}n_{O}^{+})}_{min}$ reaching a well‐defined minimum we have characterised. This ${{(}^{2}n_{O}^{+})}_{min}$ breaks planarity when using dynamically electron correlated methods (XMS‐CASPT2), which results in a drastic 1 eV decrease of the potential energy barrier required to access the ${{(}^{2}n_{O}^{+}{/}^{2}\pi_{H}^{+})}_{CI}$ and that mediates the population transfer to the cationic ${}^{2}\pi_{H}^{+}$ ground state and its minimum.

Enol‐isocytosine^+^, on the other hand, features a ${}^{2}\pi_{H-2}^{+}$ state that upon population relaxes to ${{(}^{2}\pi_{H-2}^{+}{/}^{2}\pi_{H-1}^{+})}_{CI}$ and subsequently to ${{(}^{2}\pi_{H-1}^{+})}_{min}$ . A small (∼0.1 eV) potential energy barrier is predicted to reach ${{(}^{2}\pi_{H-1}^{+}{/}^{2}n_{N}^{+})}_{CI}$ , which then further relaxes to ${{(}^{2}n_{N}^{+}{/}^{2}\pi_{H}^{+})}_{CI}$ reaching the cationic ground state.

At a difference with its keto tautomer, dynamic electron correlation appears to be negligible in enol‐isocytosine^+^ to describe both energies and geometrical structures. We thus predict an ultrafast sequential ${}^{2}\pi_{H-2}^{+}\to^{2}\pi_{H-1}^{+}\to^{2}n_{N}^{+}\to^{2}\pi_{H}^{+}$ decay for this system, similar to those previously characterised for cytosine.^[30]^

Our simulations suggest keto‐isocytosine^+^ is expected to feature longer decay lifetimes and thus may be less photostable than its enol form as well as its canonical (cytosine) counterpart.^[30]^ Moreover, the differences encountered here suggest isocytosine might be less stable to VUV photoionisation than it is to UV photo‐excitation, where it has been suggested to display similar relaxation times to those recorded for cytosine.^[19]^ We hypothesise this lesser photostability in the cationic manifold might may have contributed towards the exclusion of isocytosine from the genetic lexicon in prebiotic times, when VUV ionising radiation exposure was prominent and unfiltered by the not yet existing ozone layer.

Computed UV/Vis ground and transient absorption signals reveal similar but shifted peaks for the common ${}^{2}\pi_{H}^{+}$ state for both keto and enol tautomers, whereas more substantial differences are observed for the other states mostly due to their different character (${}^{2}n_{O}^{+}$ for keto and ${}^{2}\pi_{H-1}^{+}$ for enol). The UV/Vis region is predicted to be an appropriate spectral window to separate contributions arising from ${}^{2}\pi_{H}^{+}$ and ${}^{2}n_{O/N}^{+}$ states by featuring a well‐separated absorption feature at ∼500 nm that appears to be a general fingerprint of pyrimidine‐based nucleobase cations,[^29^, ^30^] and that could be employed to monitor their photoionisation.

Our study sheds some light on the photoionisation of non‐canonical nucleobase isocytosine, and serves as a starting point to understand the different behaviour displayed by larger DNA/RNA aggregates from a bottom‐up approach: recent results in the literature point towards a sizeable generation of cationic species in DNA even when using radiation sources below the onset of ionisation of the monomeric species.^[12]^ We expect the cationic decay pathways characterised here for monomeric species remain relevant for this process, similar to how monomer‐based de‐excitations largely contribute in DNA UV‐photoinduced phenomena.[^1^, ^2^] This work follows previous studies on canonical DNA/RNA nucleobases[^29^, ^30^] and further contributes towards understanding the intrinsic photo‐protection mechanisms of DNA/RNA and derivatives under less explored (ionising) radiation exposure.

## Supporting Information

The following are provided: Cartesian coordinates of all structures reported, tables with detailed vertical ionisation potential estimates and CASSCF vs CASPT2 bond length estimates for the different critical structures characterised.

## Conflict of interest

The authors declare no conflict of interest.

## Supporting information

As a service to our authors and readers, this journal provides supporting information supplied by the authors. Such materials are peer reviewed and may be re‐organized for online delivery, but are not copy‐edited or typeset. Technical support issues arising from supporting information (other than missing files) should be addressed to the authors.

Supporting Information
