# Supplementary material for: Modelling Photoionisation in Isocytosine: Potential Formation of Longer‐Lived Excited State Cations in its Keto Form
Source: Chemphyschem. 2021 Sep 7;22(21):2172–81. doi: 10.1002/cphc.202100402 (PMC8597144; doi:10.1002/cphc.202100402)
Supplement: Supplementary file 1 — Supporting Information [file CPHC-22-2172-s001.pdf]

ChemPhysChem

Supporting Information

Modelling Photoionisation in Isocytosine: Potential Formation of Longer-Lived Excited State Cations in its Keto Form

Javier Segarra-Martí* and Michael J. Bearpark*

CONTENTS

Table S1. Cartesian coordinates (in Å) of the key structures optimised for keto-isocytosine^{+}.
Table S2. Cartesian coordinates (in Å) of the key structures optimised for enol-isocytosine^{+}.
Table S3. Vertical ionisation potentials (in eV) of the four lowest-lying cationic states of keto-isocytosine computed with different zeroth-order CASPT2 Hamiltonians, IPEA values being provided in a.u.
Table S4. Vertical ionisation potentials (in eV) of the four lowest-lying cationic states of enol-isocytosine computed with different zeroth-order CASPT2 Hamiltonians, IPEA values being provided in a.u.
Figure S1. Minima (min) and conical intersections (CI) characterised for the lowest-lying cationic states of keto-isocytosine. All bond lengths are given in Å. CASSCF values in black; XMS-CASPT2 values in red.
Figure S2. Minima (min) and conical intersections (CI) characterised for the lowest-lying cationic states of enol-isocytosine. All bond lengths are given in Å. CASSCF values in black; XMS-CASPT2 values in red.
Figure S3. Comparison between the UV/Vis absorption of the different minima in keto- and enol-isocytosine^{+} arising from their CASSCF and CASPT2 optimised structures.
Figure S4. Comparison between the UV/Vis absorption of the $^{2}n_{N}^{+}$ minima in keto- and enol-isocytosine^{+} arising from their CASPT2 optimised structures.
Figure S5. Frequencies of the main IR fingerprints predicted for keto-isocytosine^{+} arising from their CASSCF optimised structures.
Figure S6. Frequencies of the main IR fingerprints predicted for enol-isocytosine^{+} arising from their CASSCF optimised structures.

Table S1. Cartesian coordinates (in Å) of the key structures optimised for keto-isocytosine^{+}.

FC

CASSCF

C	-0.90124672	-0.02376838	-0.28422836
N	-0.96656379	-0.05793062	0.99486003
C	0.25101240	-0.09085772	1.66223309
C	1.45050250	-0.06503478	1.07288869
C	1.52113514	-0.02312174	-0.38443746
N	0.26868257	0.00139235	-0.98826333
O	2.54012544	-0.01691949	-1.06383288
N	-2.04925791	-0.06108726	-1.05129677
H	0.16869549	-0.13263721	2.73963097
H	2.37288593	-0.08810732	1.62957054
H	0.25049314	-0.06604668	-1.98675522
H	-2.86964929	0.07815365	-0.48959661
H	-2.03681489	0.54597517	-1.85076269

XMS-CASPT2

C	-0.90978791	-0.02475329	-0.29321699
N	-0.98633868	-0.06023654	1.01873080
C	0.23055492	-0.09428536	1.67473263
C	1.46478323	-0.06563044	1.06470853
C	1.55583452	-0.02418421	-0.38342134
N	0.27014177	-0.01019759	-0.98656469
O	2.56867878	-0.01003002	-1.08727905
N	-2.06929513	-0.07610754	-1.05987654
H	0.16281181	-0.13470666	2.76733979
H	2.39098812	-0.08683249	1.64108572
H	0.26709347	-0.05902679	-2.00869430
H	-2.88791922	0.10432084	-0.47154617
H	-2.05754568	0.54168009	-1.87598839

$(^{2}\pi_{H-1}{}^{+}/^{2}n_{N}{}^{+})_{CI}$

CASSCF

C	-0.90896126	0.04357408	-0.26000704
N	-0.95322515	-0.02508630	1.03217835
C	0.23920544	-0.09918984	1.69520171
C	1.45567260	-0.10443774	1.07215543
C	1.49057639	-0.02719066	-0.36687212
N	0.28190708	0.04492732	-0.98165852
O	2.50409942	-0.01889400	-1.11810292
N	-2.02195996	0.11673383	-0.98411197
H	0.16793345	-0.15535590	2.77137488
H	2.38714720	-0.16408968	1.61101589
H	0.29240323	0.09897949	-1.99046913
H	-2.90445996	0.11812589	-0.49265295
H	-2.03033848	0.17191352	-1.98804160

XMS-CASPT2

C	−0.9061819408	0.0499245709	−0.3728450022
N	−1.0471185788	−0.0120225132	0.9186974959
C	0.0930565696	−0.0799986425	1.6980004351
C	1.3747916068	−0.0960450338	1.1898287895
C	1.6034956169	−0.0361315756	−0.2412921761
N	0.3587522704	0.0605911796	−0.9951553760
O	2.6281672147	−0.0564966678	−0.8984306210
N	−1.9656172104	0.1052635340	−1.2207160315
H	−0.0849036727	−0.1245089030	2.7761259593
H	2.2491635378	−0.1632660936	1.8405002289
H	0.4809754033	0.1124230234	−2.0169369244
H	−2.9066447392	0.0984339832	−0.8120718223
H	−1.8685020713	0.1597297457	−2.2359148718

$(^{2}n_{N}^{+})_{min}$

XMS-CASPT2

C	−0.9003126965	0.0496428609	−0.3711919524
N	−1.0930638205	−0.0100279247	0.9098388795
C	0.0556476187	−0.0987161869	1.6835050726
C	1.3514347725	−0.1221356555	1.2051447372
C	1.6123128051	−0.0482135712	−0.2139641994
N	0.3792512652	0.0200270815	−0.9928612094
O	2.6437649118	−0.0250562017	−0.8680576038
N	−1.9266869576	0.1510345102	−1.2621207696
H	−0.1288812231	−0.1480975190	2.7599703958
H	2.2119241102	−0.1785002499	1.8744271566
H	0.5231890219	0.0702820426	−2.0134743222
H	−2.8805650686	0.1809700834	−0.8799418994
H	−1.7982718832	0.1992546341	−2.2752882515

$(^{2}n_{N}^{+}/^{2}n_{O}^{+})_{CI}$

CASSCF

C	−0.91000122	0.04426209	−0.34167759
N	−0.86314203	−0.02654778	0.97182831
C	0.27483520	−0.10097217	1.72853511
C	1.47591800	−0.10451638	1.08859650
C	1.49416462	−0.03030618	−0.34447455
N	0.26567520	0.04251513	−0.99215387
O	2.49702585	−0.02323365	−1.06288850
N	−2.05764968	0.11010002	−0.99373628
H	0.14281160	−0.15193695	2.79758582
H	2.41134107	−0.16093230	1.61973545
H	0.28146538	0.09489166	−1.99880381
H	−2.91913146	0.12496523	−0.48099622
H	−2.09331253	0.18172128	−1.99154037

XMS-CASPT2

C	-0.9231288966	0.0507379852	-0.3776180463
N	-0.9011212944	-0.0180087151	0.9400296365
C	0.2087186411	-0.0745521145	1.7393498062
C	1.4416603884	-0.0723572295	1.1358141276
C	1.5405397402	-0.0323447921	-0.3201665320
N	0.3046799625	0.0742835001	-1.0043107589
O	2.5649729182	-0.0814233543	-0.9926162761
N	-2.0581304748	0.0892044976	-1.0816102767
H	0.0442601693	-0.1153592379	2.8201907526
H	2.3633848846	-0.1236526298	1.7185914235
H	0.3604644071	0.0852243713	-2.0280336641
H	-2.9497469903	0.1118623518	-0.5936120345
H	-2.0543697488	0.1802136358	-2.0930501078

$(^2n_O{}^+)_{min}$

CASSCF

C	-0.88777750	0.04349323	-0.32067307
N	-0.92644451	-0.02689371	1.01864063
C	0.20765868	-0.09679944	1.66852259
C	1.48387924	-0.10276248	1.05004757
C	1.46707668	-0.03110252	-0.30494470
N	0.27684985	0.04030138	-0.98209530
O	2.54355280	-0.02239854	-1.09236548
N	-2.03031306	0.11483104	-0.98171905
H	0.13917191	-0.15225304	2.74706221
H	2.40223763	-0.16042891	1.60946847
H	0.28920315	0.09392518	-1.98744725
H	-2.87894777	0.11989191	-0.44641875
H	-2.08614710	0.18020591	-1.97806787

XMS-CASPT2

C	-0.8905213319	0.0491728982	-0.3684342526
N	-1.0099195164	-0.0153201157	0.9584709091
C	0.1318847713	-0.0961689658	1.6658216023
C	1.4288825316	-0.1139616271	1.1306654170
C	1.4996508818	-0.0363850242	-0.2703065544
N	0.3483381277	0.0346944952	-1.0008916788
O	2.6132867447	-0.0225578583	-0.9364711695
N	-1.9852998126	0.1274007888	-1.1299758828
H	0.0081313912	-0.1510425927	2.7528756056
H	2.3342523996	-0.1749948295	1.7340431932
H	0.4202842646	0.0869197247	-2.0192984392
H	-2.8812948507	0.1283934063	-0.6466201870
H	-1.9703672090	0.1819770220	-2.1427390220

$(^2n_O{}^+/^2\pi_H{}^+)_{CI}$

CASSCF

C	-0.84587728	0.04386485	-0.30826721
N	-0.92314704	-0.02528584	1.02318052
C	0.18807022	-0.09805300	1.68952992
C	1.50998338	-0.10950759	1.10806469
C	1.47949232	-0.03506758	-0.18855589
N	0.32977988	0.04399155	-0.96137276
O	2.22959351	0.00450507	-1.38332657
N	-1.96288419	0.11378103	-0.99953371
H	0.10031083	-0.15211920	2.76668146
H	2.40229253	-0.16950804	1.70265463
H	0.31591082	0.09170997	-1.97296873
H	-2.82209698	0.11474681	-0.47843085
H	-2.00142800	0.17695197	-1.99764551

XMS-CASPT2

C	-0.9014427039	0.0496970986	-0.3715102146
N	-1.0560939253	-0.0110809961	0.9062747884
C	0.0988895070	-0.0531454269	1.6623778976
C	1.3944699818	-0.1205069307	1.2053758860
C	1.4593711129	-0.0672139486	-0.1873528330
N	0.4020913409	0.0519281122	-0.9844055031
O	2.6092107381	-0.1641354732	-0.8845993073
N	-1.8953893839	0.2708141263	-1.2812952006
H	-0.0619918530	-0.0543178239	2.7453656736
H	2.2728884428	-0.1892499000	1.8437410816
H	0.4692559919	0.2738201444	-1.9801461750
H	-2.8109801502	0.3005022796	-0.8298604170
H	-1.8963292844	-0.3181246344	-2.1134762144

$(^2\pi_{H^+})_{min}$

CASSCF

C	-0.87022809	0.04819887	-0.36271874
N	-0.93042222	-0.02093215	1.01566391
C	0.19276938	-0.09429692	1.68181251
C	1.46849186	-0.10608269	1.06307917
C	1.53658681	-0.03125296	-0.37605767
N	0.27530718	0.04500992	-1.02343955
O	2.53734368	-0.02801894	-1.04083722
N	-2.02671711	0.11716403	-0.96197991
H	0.11064000	-0.14733022	2.75914142
H	2.38666953	-0.16558277	1.62486614
H	0.29809278	0.09652231	-2.02862283
H	-2.85576365	0.11524127	-0.39030191
H	-2.12277015	0.17137026	-1.96059533

XMS-CASPT2

C	-0.8802746602	0.0486810819	-0.3655483174
N	-0.9729114078	-0.0192527559	1.0106714820
C	0.1689557460	-0.0969118085	1.6885426626
C	1.4502728841	-0.1264717714	1.0820134251
C	1.5646128880	-0.0328254771	-0.3685595209
N	0.2965938417	0.0445684277	-1.0296562592
O	2.5928799278	-0.0137195296	-1.0128540445
N	-2.0423716314	0.1229850194	-1.0008380382
H	0.0751774677	-0.1398474336	2.7788241676
H	2.3722090053	-0.1975490932	1.6639854981
H	0.3395015729	0.0920662639	-2.0517752786
H	-2.8862260438	0.1241113636	-0.4282102040
H	-2.1257655297	0.1721978780	-2.0137291158

Table S2. Cartesian coordinates (in Å) of the key structures optimised for enol-isocytosine^{+}.

FC

CASSCF

C	-1.01316562	0.00395098	-0.33570610
N	-1.14231500	0.07378778	0.98087300
C	-0.01275065	0.03627162	1.68675705
C	1.24326365	-0.06591088	1.10222650
C	1.24533210	-0.12276402	-0.29317612
N	0.14427749	-0.08896737	-1.00625235
O	2.41322513	-0.21146111	-0.93711967
N	-2.16522113	-0.02851051	-1.09348232
H	-0.11576943	0.09372983	2.76220571
H	2.15623510	-0.09363627	1.67258952
H	2.25363988	-0.25189156	-1.87271565
H	-2.96197026	0.36517677	-0.63085031
H	-2.04477128	0.29022474	-2.03534927

XMS-CASPT2

C	-1.01540517	0.01082031	-0.34362412
N	-1.16407434	0.08061028	0.99272293
C	-0.01165614	0.03784766	1.69949072
C	1.25258326	-0.07087544	1.11433819
C	1.25673471	-0.12295562	-0.29103312
N	0.14803825	-0.08379599	-1.03160411
O	2.44599001	-0.21445653	-0.93816950
N	-2.17365991	-0.02973793	-1.10126025
H	-0.11406433	0.09593730	2.78916078
H	2.17520649	-0.10327980	1.69366536
H	2.23721835	-0.24723607	-1.89377675
H	-2.98492500	0.36607927	-0.62413295
H	-2.05197618	0.28104256	-2.06577718

$(^2\pi_{H-2}{}^{+}/^2\pi_{H-1}{}^{+})_{CI}$

CASSCF

C	-0.96053315	0.07743072	-0.32442038
N	-1.17310385	0.12720257	0.98247301
C	-0.05289713	0.04360785	1.72241512
C	1.24887733	-0.08826668	1.09232369
C	1.24654685	-0.11897845	-0.36008429
N	0.16774359	-0.03768573	-1.05070292
O	2.42809469	-0.23439712	-0.92773222
N	-2.13666287	0.16234555	-1.06074458
H	-0.16165647	0.07848163	2.79552403
H	2.16410103	-0.14943568	1.65495243
H	2.36412408	-0.24821322	-1.87770005
H	-3.02334187	0.25381579	-0.56781866
H	-2.11128224	0.13409276	-2.07848516

XMS-CASPT2

C	-1.0152917403	0.0040411477	-0.3362708872
N	-1.2132199674	0.0772458171	0.9715038083
C	-0.0764793263	0.0775505576	1.7190082177
C	1.1916653652	-0.1518250060	1.1310772014
C	1.2847398596	-0.1022614878	-0.2844307648
N	0.1593137251	0.1639639043	-0.9859872826
O	2.4555180476	-0.1645246249	-0.8753455434
N	-2.1066878434	-0.2689796066	-1.1841001645
H	-0.1798141594	0.2806391613	2.7891540738
H	2.1065336446	-0.0704902967	1.7273155699
H	2.3402485331	-0.0290028486	-1.8355785940
H	-2.9850493844	0.2082293808	-0.9547960366
H	-1.8832779138	-0.3284503408	-2.1820488664

$(^{2}\pi_{H-1}^{+})_{min}$

CASSCF

C	-1.04879934	0.08467462	-0.35204728
N	-1.13458491	0.12491236	0.97396554
C	0.02741182	0.03720684	1.68869867
C	1.28209012	-0.09055176	1.12930286
C	1.29692229	-0.12012002	-0.25734922
N	0.12476971	-0.03362958	-1.01997731
O	2.37965006	-0.22817164	-0.92272558
N	-2.14419815	0.16268017	-1.09457207
H	-0.09659831	0.07501342	2.76289962
H	2.18885916	-0.15638574	1.70718856
H	2.24888144	-0.23569635	-1.87102347
H	-3.03859063	0.24915361	-0.65164792
H	-2.08580327	0.13091405	-2.09271241

XMS-CASPT2

C	-1.0512303955	0.0055652888	-0.3458177098
N	-1.1506827437	0.0752715910	1.0023462163
C	0.0274423375	0.0349598166	1.7063510062
C	1.2921008412	-0.0694415317	1.1368804623
C	1.2966709088	-0.1171492966	-0.2713765403
N	0.1216597230	-0.0800398982	-1.0602294085
O	2.3866751189	-0.1965083968	-0.9505810258
N	-2.1678671801	0.0211709736	-1.0952018647
H	-0.0887428380	0.0927057652	2.7931047874
H	2.2201776113	-0.0992181800	1.7087609867
H	2.1686470596	-0.2150033591	-1.9094645546
H	-3.0742934823	0.0889969367	-0.6361720061
H	-2.1044285983	-0.0184681855	-2.1087099900

$(^2\pi_{H-1}{}^+/^2n_N{}^+)_{CI}$

CASSCF

C	-1.03639845	0.07959297	-0.33865987
N	-1.16773002	0.12508049	0.98687970
C	0.02390325	0.03496830	1.67745296
C	1.28617733	-0.08288043	1.13282378
C	1.31204651	-0.12023726	-0.24739018
N	0.11881194	-0.04393915	-1.05551366
O	2.37140436	-0.22522620	-0.92452515
N	-2.13118180	0.15598350	-1.09254796
H	-0.08852337	0.06560181	2.75347522
H	2.18736573	-0.14509053	1.72035621
H	2.21838581	-0.23949741	-1.87228888
H	-3.02393651	0.25853698	-0.64986725
H	-2.07031477	0.13710692	-2.09019492

XMS-CASPT2

C	-1.0274966953	0.0045587540	-0.3395130380
N	-1.2040317198	0.0787343257	1.0133668988
C	0.0106528586	0.0233576492	1.6976150615
C	1.2882853420	-0.0900254653	1.1570831150
C	1.3150372173	-0.1174356119	-0.2468916361
N	0.1288708701	-0.0845344249	-1.0996243669
O	2.3788979033	-0.1657948582	-0.9406713155
N	-2.1321625793	0.0160329290	-1.1134905386
H	-0.1038920746	0.0820534888	2.7834376415
H	2.2071542824	-0.1213258707	1.7433025628
H	2.1258093980	-0.1714012198	-1.8974644805
H	-3.0436993488	0.0791311407	-0.6614809272
H	-2.0577323654	-0.0210903084	-2.1264503096

$(^2n_N{}^+/^2\pi_H{}^+)_{CI}$

CASSCF

C	-1.08024348	0.07412484	-0.39241175
N	-1.02584053	0.10454121	0.91968819
C	0.05629273	0.03451437	1.72450632
C	1.28579723	-0.08232997	1.14331853
C	1.25885534	-0.11622223	-0.27646430
N	0.13138741	-0.03947868	-0.95155887
O	2.38720525	-0.22736205	-0.91619650
N	-2.18589404	0.13372797	-1.10460900
H	-0.12746705	0.07580467	2.78820718
H	2.20769874	-0.14576680	1.69621954
H	2.28944588	-0.24619231	-1.86438255
H	-3.07271946	0.27425092	-0.66284117
H	-2.12450804	0.16038808	-2.10347563

XMS-CASPT2

C	-1.0892815098	0.0071790183	-0.3559256905
N	-1.0381114660	0.0684795273	0.9898934009
C	0.0774967517	0.0417702922	1.7250533679
C	1.3152395759	-0.0566258326	1.0933744166
C	1.2552430329	-0.1209949748	-0.3328892226
N	0.0963696615	-0.0891011489	-0.9978953950
O	2.3892502069	-0.2146190787	-0.9986773919
N	-2.2441427245	0.0397253696	-1.0201439332
H	-0.0404557072	0.0993808080	2.8131261621
H	2.2634551452	-0.0829870545	1.6328461008
H	2.1994336445	-0.2489973321	-1.9550820626
H	-3.1253260959	0.1140736945	-0.5189289391
H	-2.2356725936	-0.0108446112	-2.0356989326

$(^2\pi_{H}{}^{+})_{min}$

CASSCF

C	-0.99918339	0.07916971	-0.36240567
N	-1.15380401	0.12382440	1.00620875
C	-0.05229672	0.04432716	1.70520587
C	1.21701024	-0.07989769	1.08575616
C	1.23643358	-0.11636378	-0.35638790
N	0.15549022	-0.03841505	-1.06265509
O	2.40963250	-0.23286762	-0.91790378
N	-2.10212089	0.16058885	-1.04416098
H	-0.14209430	0.07636820	2.78228070
H	2.13583110	-0.14844661	1.64548368
H	2.35557938	-0.25088654	-1.87004960
H	-2.98353553	0.24803734	-0.55745342
H	-2.07693221	0.13456162	-2.05391872

XMS-CASPT2

C	−1.0063822306	0.0036632997	−0.3339041457
N	−1.1470720252	0.0768715046	1.0401011326
C	−0.0010812894	0.0542853859	1.7166546832
C	1.2572627024	−0.0395413717	1.0707986991
C	1.2472641180	−0.1272868327	−0.3731901443
N	0.1262721205	−0.1020534569	−1.0806366913
O	2.4130589583	−0.2314678225	−0.9627132617
N	−2.1603646553	0.0415792925	−0.9967058394
H	−0.0671375388	0.1136787983	2.8075412815
H	2.2037420690	−0.0436701681	1.6164231495
H	2.2689357861	−0.2803190412	−1.9290603535
H	−3.0320248793	0.1202115888	−0.4705378637
H	−2.1657835362	−0.0042927363	−2.0166259569

Table S3. Vertical ionisation potentials (in eV) of the four lowest-lying cationic states of keto-isocytosine computed with different zeroth-order CASPT2 Hamiltonians, IPEA values being provided in a.u.

		$^2\pi_H{}^+$	$^2n_O{}^+$	$^2n_N{}^+$	$^2\pi_{H-1}{}^+$
CASSCF		6.37	8.05	8.48	8.98
CASPT2	IPEA = 0.0	8.48	9.75	10.32	10.25
	IPEA = 0.25	8.67	9.92	10.49	10.57
MS-CASPT2	IPEA = 0.0	8.51	9.83	10.41	10.34
	IPEA = 0.25	8.71	9.99	10.66	10.55
XMS-CASPT2	IPEA = 0.0	8.77	9.95	10.55	10.82
	IPEA = 0.25	8.96	10.23	10.81	11.10
CASPT2 Average		**8.68**	**9.95**	**10.54**	**10.60**

Table S4. Vertical ionisation potentials (in eV) of the four lowest-lying cationic states of enol-isocytosine computed with different zeroth-order CASPT2 Hamiltonians, IPEA values being provided in a.u.

		$^2\pi_H{}^+$	$^2n_N{}^+$	$^2\pi_{H-1}{}^+$	$^2\pi_{H-2}{}^+$
CASSCF		7.71	9.06	9.82	10.64
CASPT2	IPEA = 0.0	8.53	9.30	10.35	10.94
	IPEA = 0.25	8.69	9.49	10.53	11.24
MS-CASPT2	IPEA = 0.0	8.66	9.44	10.44	11.10
	IPEA = 0.25	8.80	9.60	10.61	10.37
XMS-CASPT2	IPEA = 0.0	9.10	9.84	10.86	10.55
	IPEA = 0.25	9.23	10.02	11.04	11.80
CASPT2 Average		**8.83**	**9.61**	**10.64**	**11.33**

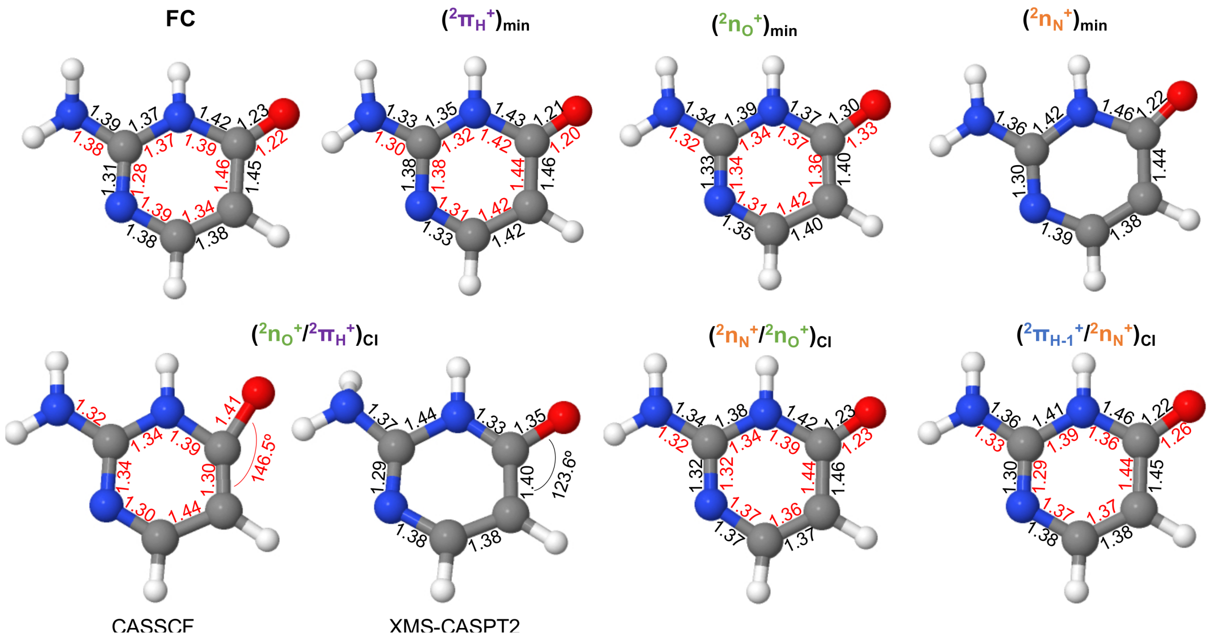

Figure S1. Minima (min) and conical intersections (CI) characterised for the lowest-lying cationic states of keto-isocytosine. All bond lengths are given in Å. CASSCF values in red; XMS-CASPT2 values in black.

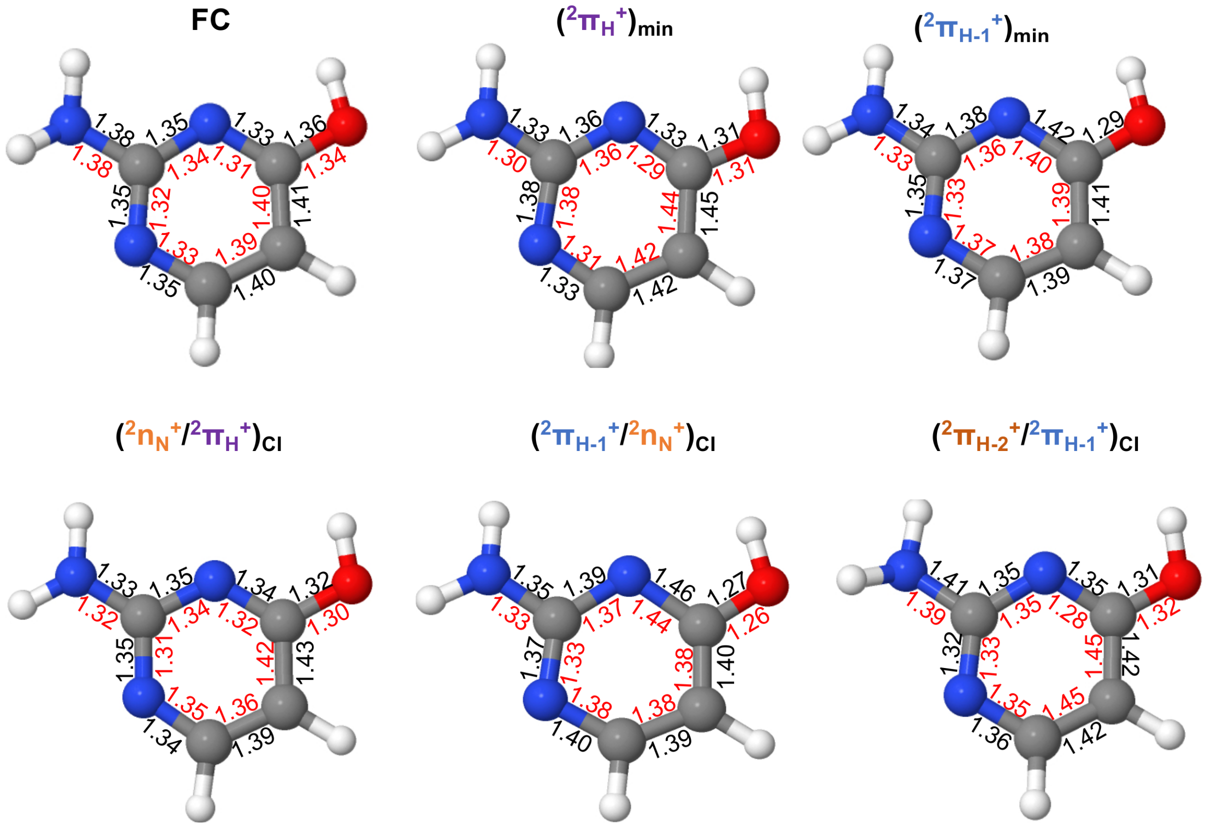

Figure S2. Minima (min) and conical intersections (CI) characterised for the lowest-lying cationic states of enol-isocytosine. All bond lengths are given in Å. CASSCF values in red; XMS-CASPT2 values in black.
